# Black phosphorous nanomaterials as a new paradigm for postoperative tumor treatment regimens

**DOI:** 10.1186/s12951-022-01579-3

**Published:** 2022-08-11

**Authors:** Yanhua Hou, Yang Fei, Zehong Liu, Yingqi Liu, Menghuan Li, Zhong Luo

**Affiliations:** 1 Chongqing Engineering Research Center of Pharmaceutical Science, Chongqing Medical and Pharmaceutical College, Chongqing, 401331 China; 2 School of Life Science, Chongqing University, Chongqing, 400044 China; 3 111 Project Laboratory of Biomechanics and Tissue Repair, College of Bioengineering, Chongqing University, Chongqing, 400044 China

**Keywords:** Black phosphorus, Postoperative tumor treatment, Wound healing, Tissue reconstruction

## Abstract

Surgery is currently a mainstream treatment modality for various solid tumor indications. However, aggressive resection of tumor tissues frequently causes postoperative complications, which severely undermine the well-being of patients. Moreover, the residue tumor cells may substantially increase the risk of local and distant tumor relapse. The recent development in black phosphorus (BP)-based nanomaterials offers a promising opportunity to address these clinical challenges. BP is an emerging nanomaterial with excellent biocompatibility and versatile functionality, which has already demonstrated great potential for a variety of biomedical applications including tumor therapy and tissue engineering. In this review, the recent advances in BP-based nanobiomaterials for the post-surgery treatment of solid tumor have been summarized, while specific emphasis was placed on their capability to continuously inhibit residue tumor growth at the surgery site as well as stimulating various healing mechanisms, aiming to preventing tumor relapse while promoting the healing of surgery-induced traumatic soft/hard tissue injuries. It is anticipated that the nanoengineered BP-based materials may open new avenues to tackle those clinical challenges in surgical treatment of solid tumors.

## Introduction

Cancer is one of the leading causes of death around the globe, which accounts for around 10 million deaths in year 2020 alone and the incidence and mortality rates are still on a rapid rise, imposing great burden on the patients, families and society [1–3]. Solid tumors represent around 90% of adult cancers and are considered a major hurdle for successful treatment [4]. Typically, solid tumor tissues are characterized by poor perfusion and high interstitial pressure, which severely limit the entry and retention of antitumor chemotherapeutics [5–7]. Meanwhile, solid tumors are constantly in a hypoxic state that significantly enhances their resistance to radiotherapy [8, 9]. Up to now, surgery remains one of the primary approaches for the treatment of common solid tumor indications [10, 11]. During a typical surgical procedure, the solid tumor tissues are removed with some of the periphery healthy tissues, which is termed “resection” and ensures that the cancerous masses are removed as much as possible [12, 13]. However, clinical evidence collectively demonstrated that solid tumors are usually highly infiltrative. Some microscopic tumor cells may remain even after aggressive resection, which could lead to tumoral seeding at the surgery site and significantly increase the risk of local recurrence [14, 15]. Moreover, the extensive removal of peri-tumor healthy tissues would substantially under the structural and functional integrity of the original organs and tissues, which may not only seriously reduce the quality of life of the solid tumor-bearing patients but also cause severe post-surgery complications such as bleeding, infection or loss of body functions [16, 17]. Consequently, the postoperative treatment of patients with solid tumor indications represents a complex challenge against current antitumor and tissue engineering technologies, warranting the development of new adjuvant therapies. These new treatments should be able to continuously inhibit the growth of tumor cells at the surgery site to reduce the risk of both local and distal recurrence as well as to facilitate wound healing and recovery of body functions.

The concept of nanotechnology has revolutionized many scientific and healthcare sectors, which provides ample opportunities to overcome the current limitations in solid tumor treatment. Interestingly, two-dimensional inorganic nanomaterials are an emerging class of biomaterials that have demonstrated significant potential for biomedical applications on account of their unique planar topography and novel physical, chemical and biological characteristics, of which the most prominent examples include graphene, boron,[18] antimonene [19], biotite [20], arsenene [21, 22], vermiculite,[23], FeOCl [24], MoS_2_ [25], WS_2_ [26] and black phosphorus (BP) [27, 28]. These novel 2D inorganic materials potentiate enhanced interaction with various drugs, biomolecules and cells as well as offering unprecedent features such as light-responsiveness, tunable surface chemistry, anisotropic mechanical behaviors and chemical reactivity, which are rarely found in conventional biomaterials. Among the wide range of 2D nanomaterials that have been developed in recent years, BP has demonstrated remarkable potential for tumor diagnosis and therapy owing to their versatile physicochemical properties and total degradability in vivo (Table 1) [29–33]. Typically, BP is the most stable allotrope of phosphorous, which is essentially constituted by multiple single-atom layers held together by non-covalent van der Waals interactions, and the lamellar structures could be exfoliated down to nanosheets with varying thicknesses via low-cost approaches [34–36]. Taking advantage of the large surface area and covalent/non-covalent interaction modes with various therapeutic substances, BPs could be implemented as robust delivery platforms for diagnostic and therapeutic applications [37–39]. Meanwhile, unlike other typical 2D nanomaterials such as graphene or MoS_2_ nanosheets, BP usually presents a tunable band gap dependent on the number of layers, ranging from a relatively large band gap of around 2 eV for single-layer BP to a small band gap of around 0.3 eV for bulk BP [40–42]. The tunable band gap range of BP nanosheets enables broad spectroscopic absorption from ultraviolet to infrared region, which could be used for triggering various light-dependent therapeutic activities including photodynamic therapy, photothermal therapy and light-actuated drug release [43, 44]. In addition to the well-elucidated phototherapeutic mechanisms, the recent advances in BP nanomaterials have revealed many more unprecedent properties, which could be used to create novel therapeutic opportunities for tumor treatment. For instance, it is recently demonstrated that BP nanosheets could generate abundant ROS under X-ray exposure, substantiating its potential application for radiosensitization of tumor cells [45, 46]. Alternatively, Yu et al. discovered that BP nanosheets could show intrinsic toxicity in cellular environment due to the spontaneous ROS generation during BP oxidation [47]. Follow-up studies further revealed that the intrinsic chemotherapeutic effect of BP nanosheets is more pronounced in tumor cells than normal cells, which may be caused by the aberrant metabolic patterns therein [48]. In addition to the versatile biomedical functions of BP nanosheets, they could also be facilely integrated with other pharmacological components or substrates to enhance their therapeutic performance or endow novel functions [49]. These therapeutically relevant properties of BP nanosheets may significantly expand our arsenal against various tumor indications while improving the treatment efficacy and persistence (Table 2). Remarkably, recent studies collectively demonstrate that BP nanomaterials presents potent inhibition effect against solid tumors and hematological tumors, suggesting their immense application potential in numerous clinical scenarios [50, 51]. Exfoliated BP nanosheets show excellent biocompatibility in pre-clinical tests and could be completely degraded into non-toxic phosphate ions in aqueous environment [52, 53]. Based on the merits stated above, BPs have attracted substantial interest for tumor therapy and tissue engineering applications, suggesting their translational potential for post-operative treatment of solid tumors.

**Table 1:** Comparison of the therapeutically relevant properties of BP with other typical 2D inorganic materials

	BP	Graphene	Antimonene	Biotite	Arsenene	Vermiculite	FeOCl	WS_2_	MoS_2_
Chemical composition	P	C	Sb	K, Al, Si, O	As	Mg, Fe, Al, Si, O	Fe, O, Cl	W, S	Mo, S
Conductivity	Semiconductive	Conductive	Semiconductive	Insulative	Semiconductive	Insulative	Insulative	Semiconductive	Semiconductive
Photothermal capability	Yes	Yes	Yes	Yes	Yes	Yes	Not reported	Yes	Yes
Photodynamic capability	Yes	Yes	Yes	Yes	Yes	Yes	Not reported	Not reported	Not reported
Biocompatibility	Good	Good	Good	Good	Good	Good	Good	Good	Good
Degradability in vivo	Completely degradable, degradation products are non-toxic	Requiring additional engineering	Degradable	Degradable	Degradable	Degradable	Not reported	Requiring additional engineering	Requiring additional engineering
Reference	[93]	[131]	[132]	[20]	[133]	[23]	[24]	[134]	[135]

**Table 2:** Summary of the therapeutically relevant properties of BP nanosheets

Chemical composition		
Element	Bond type	Bond length
P atoms	Covalent binding between sp^3^ hybridized P atoms	0.2224 nm (between nearest P atoms in the same plane); 0.2244 nm (between top and bottom P atoms in the same layer)
Atomic topography		
Atomic configuration	Single layer thickness	Interlayer distance
Puckered honeycomb pattern	Around 0.85 nm	Around 0.53 nm
Drug delivery capability		
Drug loading mode	Drug loading potential	Drug release sensitivity
Covalent binding, electrostatic interaction, π-π interactions, hydrophobic interactions	High drug loading potential due to abundant binding sites and large surface-area-to-volume ratio	Drug release could be triggered by heating, light, acidity, ultrasound, etc.
Therapeutic capability		
PTT	PDT	Inherent bioactivity
Good photothermal potential under NIR illumination	Good quantum yield at around 0.91 under irritation at 660 nm for efficient ROS generation	Inducing tumor specific toxicity through ROS generation and cell cycle arrest
Degradation behavior		
Degradation mechanism	Degradability	Degradation product
Formation of P-O-P bonds, followed by the attack of water molecules and removal of P atoms	Completely degradable in vivo, degradation can be accelerated in the presence of light, heating, basic pH and oxygen	Non-toxic phosphate and phosphonate ions

In 2014, Liu et al. and Li et al. first reported a mechanical exfoliation method for obtaining few-layer BP nanosheets, which has inspired a surge in the research and application of BP-based nanomaterials [54, 55]. The research effort in related areas in the past decade has significantly expanded our knowledge regarding the preparation strategies, fundamental properties and biomedical applications of BP-based nanomaterials, which have been extensively reviewed in previous publications. However, the application of BP for postoperative care after solid tumor surgery has not been systematically reviewed despite the burgeoning development in this field in recent years. In this review, we first provided a concise yet comprehensive discussion regarding the unique functional properties of BP-based nanomaterials as well as the recent advances in BP preparation technologies for biomedical applications, followed by a thorough analysis regarding the application of BP-based nanobiomaterials for the postoperative care of solid tumors, which included (1) wound care after soft tissue resection and (2) wound care after hard tissue resection. The existing challenges and future opportunities of BP-based nanomaterials were also covered by referring to the latest reports in this area. It is anticipated that the concepts and insights in this review may facilitate the clinical translation of BP-based nanomaterials as adjuvant therapies for patients following solid tumor resection.

## Toxicity of BP-based biomaterials

The intriguing physical and chemical properties of BP nanosheets offer novel solutions for various biomedical applications, which also raise the need to comprehensively investigate their toxicity in cellular, tissue and systemic levels. According to an early study by Latiff et al., the cytotoxicity of layered BP nanosheets to A549 cells is dose-dependent, for which the cell viability drops to around 34% under the BP dose of 50 µg/mL for 24 h. Comparative analysis further shows that the cytotoxicity of BP nanosheets is generally higher than those 2D nanoscale transition-metal dichalcogenides such as MoS_2_, WS_2_ and WSe_2_ but lower than graphene oxide [56]. Recent studies reveal more interesting details regarding the cytotoxic characteristics of BP nanosheets. For instance, Zhao et al. discovered that the length scale and thickness of the BP nanosheets could also exert profound impact on their toxicity profiles, where larger and thicker BP nanosheets tend to demonstrate higher toxicity than the smaller ones. Mechanistic evaluations confirm that the size-dependent BP cytotoxicity is due to the enhanced ROS generation and cell membrane disruption under larger sizes [57]. Meanwhile, it is also demonstrated that the cytotoxic impact of BP nanosheets is affected by the cell types, which show significantly higher cytotoxicity to 293 T cells compared to NIH 3T3 and HCoEpiC cells [58]. Interestingly, due to the aberrant energy metabolism in tumor cells, they tend to have much higher uptake rate of BP-based biomaterials compared to normal cells, leading to the generation of abundant lipid peroxides and suppressed superoxide dismutase activities. Consequently, tumor cells are usually more susceptible to BP-mediated cytotoxic effect even without light stimulation [44]. Similarly, Zhou et al. also reported that due to the intrinsically higher oxidative stress and energy metabolism rate in tumor cells, they are more susceptible to the G2/M phase arrest induced by the spontaneous degradation of BP nanosheets, leading to greater apoptosis/autophagy-dependent tumor inhibition effect [48]. Furthermore, it is reported that BPs could inhibit the PLK1 kinase in cells to cause prolonged mitosis arrest, eventually leading to apoptotic cells death. As PLK1 is usually upregulated in various tumor cell lines, the BP treatment may eliminate tumor cells with relatively high selectivity while sparing normal cells [59]. It is also notable that exfoliated few-layer BP nanosheets are prone to oxidative degradation during preparation and storage and generate phosphorus oxide groups on BP surface, which would not significantly affect the toxicity profile of BP-based biomaterials [56]. Overall, these studies collectively demonstrate the low systemic toxicity of BP nanosheets as well as their inherent tumor-selective inhibition effect under clinically relevant conditions, although comprehensive and systemic safety evaluations are still warranted to guide the translation and application of BP-based biomaterials.

## Degradation mechanism of BP nanosheets

**Fig. 1:**
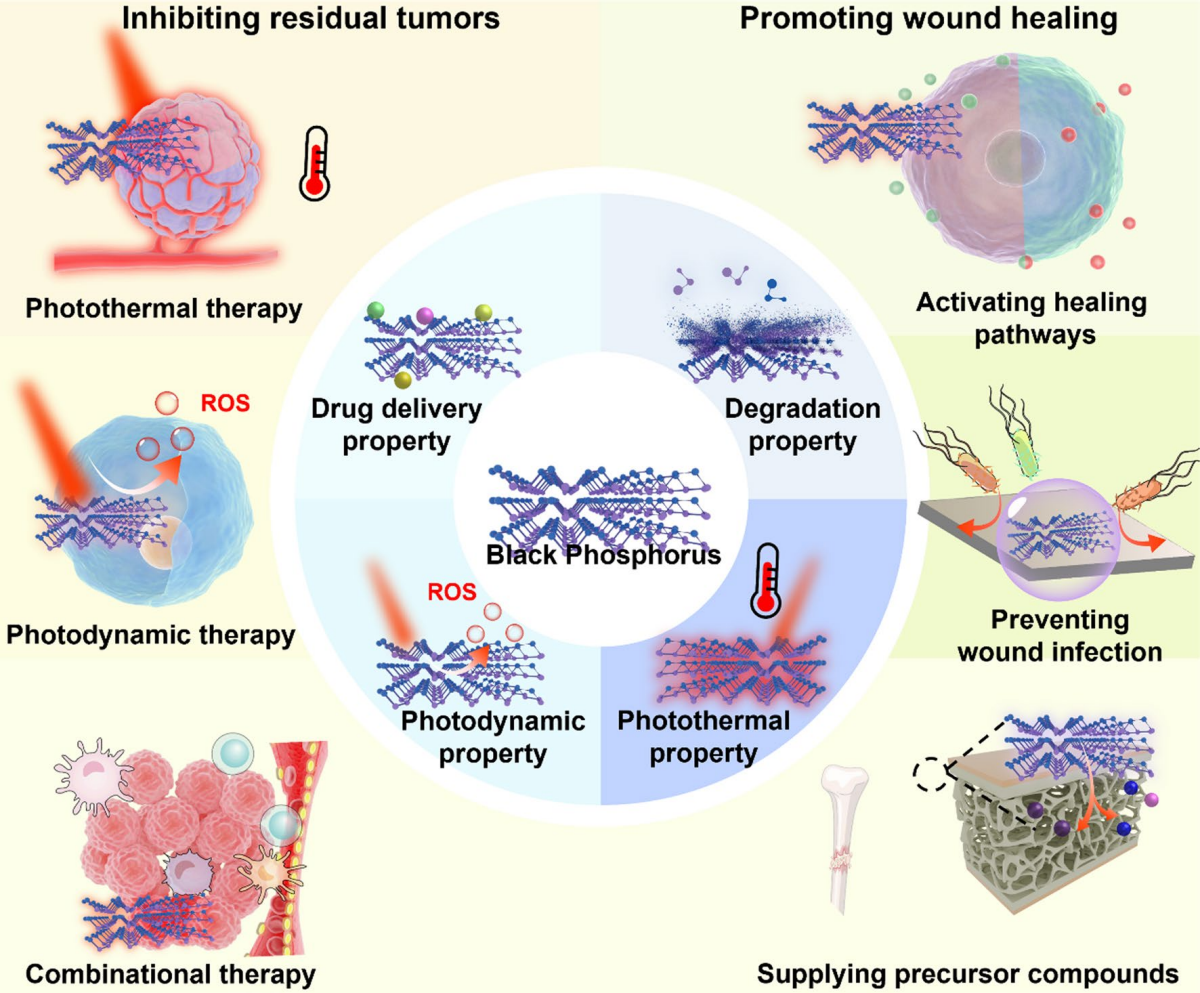
Schematic illustration of the fundamental functional properties of black phosphorus nanomaterials and their therapeutic relevance for the postoperative treatment of resected solid tumors

Bulk BP is thermodynamically stable and chemically inert. However, after being exfoliated into the few-layer nanoscale form, BP becomes thermodynamically unstable and shows high susceptibility to oxidation in aqueous environment. From a mechanistic perspective, the oxidation of black phosphorus refers to the process that ambient oxygen atoms are inserted into the P-P bonds to form P-O-P bonds, eventually forming phosphate or phosphonate ions and thus being detached from the BP layers [60]. Although much of the BP oxidation process is still poorly understood, previous studies have confirmed water and oxygen are the two crucial factors to initiate BP oxidation. Specifically, as the P atoms in BP layers are sp^3^ hybridized, each P atom would carry a lone pair of electrons and thus render strong reducibility. Oxygen and water in the ambient environment could react with the P atoms to form complex phosphorus oxides. Oxidation of BP usually initiates at structurally defective sites and edges, which have much lower energy barrier (around 6 eV) compared to undamaged BP surface (around 10 eV). There are generally two steps for BP oxidation, for which (1) the oxygen atoms are firstly chemisorbed on the BP surface to form oxidized phosphorene, followed by the (2) exothermic reaction between water molecules and oxidized phosphorene [24]. Interestingly, neither dry oxygen or deoxygenated water could induce significant BP oxidation, suggesting potential synergy between the two oxidative factors that remains to be explored [61]. Meanwhile, it is confirmed that light irritation could accelerate the BP degradation process. According to the study by Zhou et al., the incident light would first excite the electrons in the conduction band of BP, which are subsequently transferred to the absorbed oxygen molecules to generate superoxide anions (O_2_^−^). The O_2_^−^ species would then be dissociated on the BP surface to form P-O-P bonds, which are further attacked by water molecules and eventually lead to the removal of P atoms [62]. These insights regarding the degradation of BP nanosheets in clinically relevant environment offers new approaches to enhance their stability in the presence of light, oxygen and water without altering electron configurations and therapeutic performance (Fig. 1).

## Applications of BP-based nanomaterials for postoperative care of tumors

### Applications on soft organ/tissue-residing tumors

Malignancies can occur in almost all soft organs and glands such as skin, liver, brain, lungs and kidneys as well as those connective tissues including muscles, fat and ligaments [63–65]. Surgery is the primary treatment for most of these tumor indications in clinical practice for both curative and palliative purposes [66–68]. However, it is well-established that tumors are highly invasive and some microscopic tumors would inevitably remain in the postoperative wound despite maximum safe resection, which may act as seeds to initiate both local relapse and distant metastasis [69, 70]. Moreover, the aggressive surgical removal of solid tumors from the peripheral normal tissues would often cause severe traumatic damage that may not only undermine their integrity and physiological functions but also elevate the risk of post-surgical complications such as bleeding and wound infection [71, 72]. The repair of the surgical wound and regeneration of the traumatic tissues have become one of the primary goals for postoperative wound care. However, the surgery treatment would severely disrupt the metabolic homeostasis and the endocrine systems, thus significantly impairing the wound healing capacities of tissues in the surgical site as well as enhancing the risk of tumor relapse [73]. To improve the healing rate of surgical wound, additional treatment is usually needed to reduce blood loss, facilitate the migration of proliferation of local normal cells, enhance deposition and remodeling of extracellular matrix, ameliorate the local inflammation status and prevent postoperative wound infection. Consequently, an ideal post-surgical treatment for tumors should be able to simultaneously inhibit the growth of residual tumor cells in the resection wound while restoring the integrity and functions of the damaged normal tissues, which are critical for improving the disease-free survival and life quality of patients. Interestingly, the versatile biofunctions of BP-based nanomaterials offer ample opportunities to address these clinical concerns. In terms of the BP-mediated elimination of residual microscopic cells, the BP nanosheets are usually (1) immobilized in functional polymeric scaffolds and administered locally into the wound bed or (2) modified with tumor-specific ligand for systemic administration. This targeted treatment approach could elicit various cytotoxic effects on the residual microscopic tumors in the resection margin to inhibit their growth and proliferation while sparing normal cells, thus reducing the risk of local tumor relapse at optimal safety. For instance, Ding et al. coated BP quantum dots with erythrocyte membrane and modified their surface with iRGD, which could home to the residual microscopic tumor cells after intravenous injection. After the uptake by tumor cells, the functional BP quantum dots could demonstrate glucose oxidase-like catalytic activity under NIR illumination and generate abundant cytotoxic ROS, leading to simultaneous photothermal/nanocatalytic therapy to inhibit tumor recurrence and prolong the postoperative survival of the mice [74]. On the other hand, the functional capabilities of BP nanosheets could be facilely exploited to improve wound healing by ameliorating local inflammation, preventing infection and promoting angiogenesis [75]. These reports evidently suggest the antitumor and pro-healing ability of BP-based biomaterials, supporting their application for the postoperative treatment of resected tumors in soft tissues (Table 3).

**Table 3:** Summary on the preparation and therapeutic activity of various BP-based preparations for postoperative treatment of tumors in solid tissues

Carrier substrate	Therapeutic additive	Tumor indication	Antitumor mechanism	Wound healing mechanism	Refs.
PDLLA-PEG‐PDLLA: PLEL hydrogel	No	Resected HeLa tumors	PTT	PTT-mediated antibacterial effect	[83]
Gelatin hydrogels	No	Resected breast cancer	PTT	PTT-enhanced formation of lipid droplets and adipogenic related genes, hydrogel facilitated migration and infiltration of normal cells	[84]
Agarose hydrogel	Stress granule inhibitor Emetine	Hepatocellular carcinoma	Emetine suppresses stress granule formation to sensitize tumors for PTT	Not mentioned	[85]
Cellulose hydrogel	No	Melanoma, hepato-carcinoma and reticulum cell sarcoma	PTT	Not mentioned	[86]
Agarose hydrogel	Doxorubicin	Breast cancer, cervical cancer, lung carcinoma, melanoma	PTT, PTT-triggered drug release	Not mentioned	[87]
Gelatin-PCL nanofibrous scaffold	Doxorubicin	Resected melanoma	PTT, PTT-triggered drug release	Release of phosphates and phosphonates, photothermal stimulation of ERK1/2 and PI3K/Akt healing pathways	[88]
BPs are first coated with tumor cell membrane then loaded into hyaluronic acid/pluronic F-127 hydrogels	PD-1 antibody, GM-CSF and lipopolysaccharide	Triple negative breast cancer	PTT-stimulated cross-presentation by APCs and PTT-triggered aPD-1 release	Not mentioned	[89]
NIPAM hydrogel	γδ T cell agonist zoledronate	Breast cancer, bladder cancer	PTT, PTT-triggered zoledronate release	Not mentioned	[90]
PPS-PAA-PEG vesicle	Ag^+^ions	Triple negative breast cancer	PDT, PDT/Ag^+^ stimulated antitumor immune responses	Ag^+^-mediated antibacterial effects	[96]

**Fig. 2:**
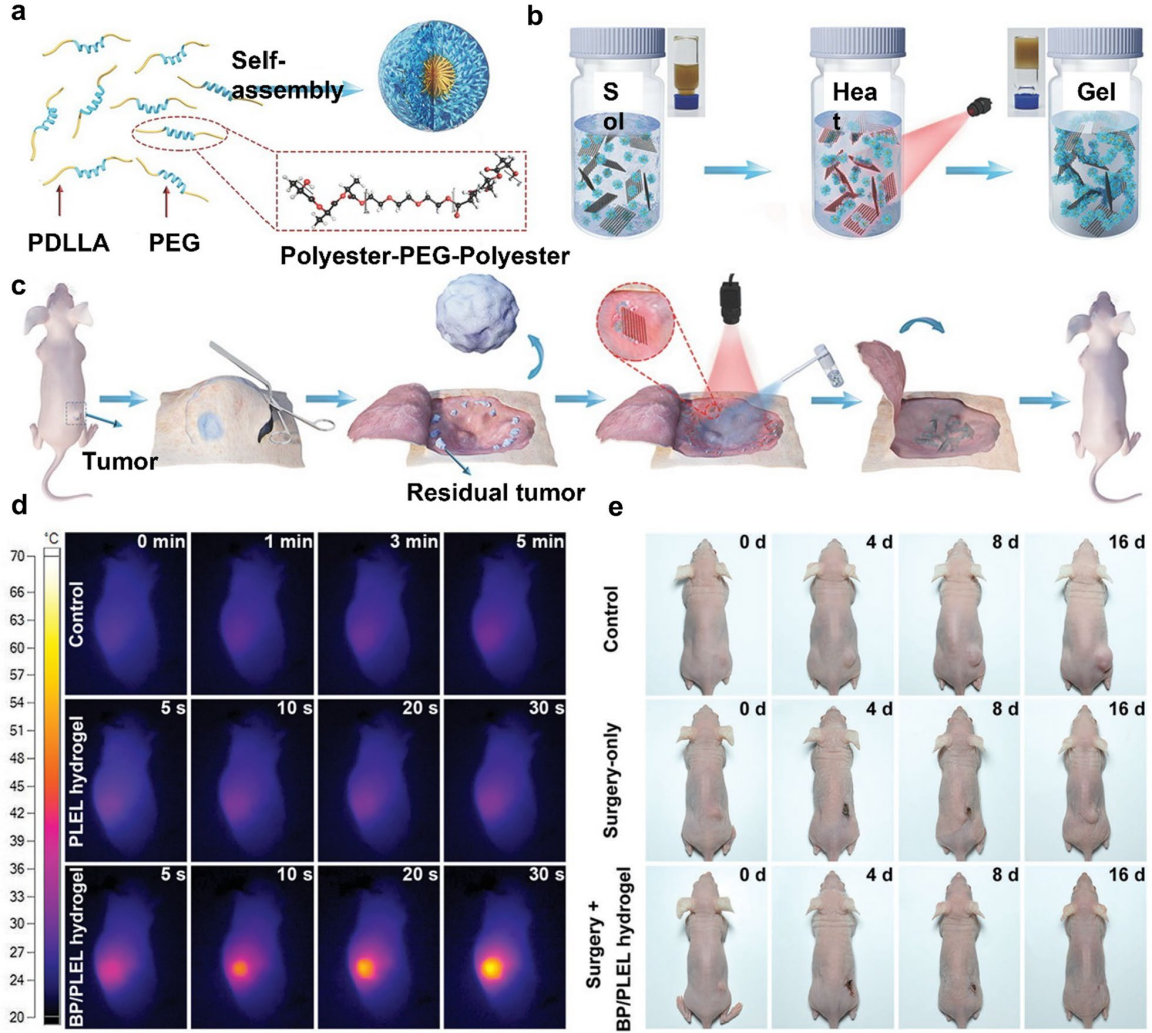
**a** Synthesis scheme of the thermosensitive micellar PDLLA-PEG-PDLLA hydrogel precursors. **b** Light-triggered formation of the BP-incorporated hydrogel. **c** Schematic illustration of the hydrogel-enabled photothermal therapy of resected solid tumors. **d** IR thermal imaging of the hydrogels under NIR illumination after implantation. **e** Photothermal-capable BP-incorporated hydrogels suppress postoperative tumor growth while enhancing wound healing. Reproduced with permission from Ref [83]. Copyright © 2018 The Authors. Published by WILEY-VCH Verlag GmbH & Co. KGaA, Weinheim

Photothermal effect is one of the most extensively studied therapeutic functions of BP-based nanomaterials. Specifically, taking advantage of the high absorption coefficient and light extinction ratio of BP nanosheets, they could efficiently convert the incident light into thermal energy to induce a significant increase in local temperature [76] .Moreover, due to the lay-dependent band gap of the BP nanosheets, their absorption peak could be tuned in the near-infrared (NIR) range, which has higher tissue penetration and lower phototoxicity compared to ultra-violet and visible light, thus adding to the efficacy and safety of the BP-mediated photothermal treatment both for skin and deeply buried organs and tissues [49, 77, 78]. The BP-mediated localized heating under NIR treatment would induce severe damage to tumor cells such as disruption of cellular membranes, denaturation of key proteins, creating DNA breaks and causing chromosomal aberrations, leading to the initiation of programmed cell death sequences including apoptosis, necrosis, etc [79, 80]. In addition to the direct tumor killing benefits, it is recently revealed that the photothermal treatment could promote blood flow in the post-surgical wounds and increase the nutrient and oxygen supply to facilitate wound repair [81, 82]. The therapeutic benefit of photothermal-capable BP-incorporated hydrogels for the postoperative care of resected tumors is excellently demonstrated in the recent report by Shao et al., in which the authors facilely incorporated BP nanosheets into thermosensitive poly(d,l-lactide)-poly(ethylene glycol)-poly(d,l-lactide (PLEL) hydrogels for the post-surgical treatment of subcutaneous tumors (Fig. 2) [83]. The BP nanosheets were firstly obtained via liquid exfoliation method and then mixed with PLEL micelles in aqueous solution. Under NIR irritation at around 808 nm of around 5 min, the temperature of the mixture solution would increase above the critical transition temperature of the PELE contents and cause the formation of mechanically stable BP-encapsulated hydrogels. Taking advantage of the remotely controllable gelling process, the BP/PLELE precursors could be stored in the liquid form and administered to the wound bed of the resected tumors via simple spraying before the NIR-mediated sol-gel-transition. In vivo analysis showed that the BP-incorporated PLEL hydrogels have excellently maintained the photothermal generation efficiency of BP nanosheets that the temperature of the hydrogel-implanted wound bed increased to more than 58 °C within only 30 s of NIR illumination under a relatively low NIR dose of 0.5 W cm^− 2^. The BP-enabled photothermal heating not only inhibited the growth of residual tumor cells in the wound bed to prevent local relapse but also effectively sterilized more than 99.5% of bacteria the wound tissues, thus significantly reducing the risk of postoperative wound infection for robust wound healing. Furthermore, the whole hydrogel system may limit the photothermal activity of BP nanosheets in the wound site and is completely degradable in biological environment, thus minimizing the safety risks in both the short and long term after implantation. Sutrisno et al. constructed BP-doped gelatin hydrogels with interconnected pore structures by adding ice particles during the gelation process [84]. The gelatin hydrogel scaffold could preserve the photothermal performance of the BP nanosheets for sustained photothermal treatment, while addition of BP nanosheets enhanced the compression strength of the polymeric network to improve its persistence in the complex physical tissue environment after implantation. The composite hydrogel demonstrated BP dose-dependent photothermal efficiency in vivo that enabled efficient elimination of residual breast cancer cells in the incision wound. Moreover, the degradation products of BP nanosheets could promote the formation of lipid droplets and elevate expression of adipogenic-related genes, while well-defined porous network in the hydrogels allowed the migration and infiltration of regeneration-related cells, leading to the accelerated reconstruction of the postoperative breast defects. Similar designs were also realized using cellulose, agarose, etc.,[85–87] which may provide modulable mechanical and biochemical performance to address the specific needs by different tumor indications.

**Fig. 3:**
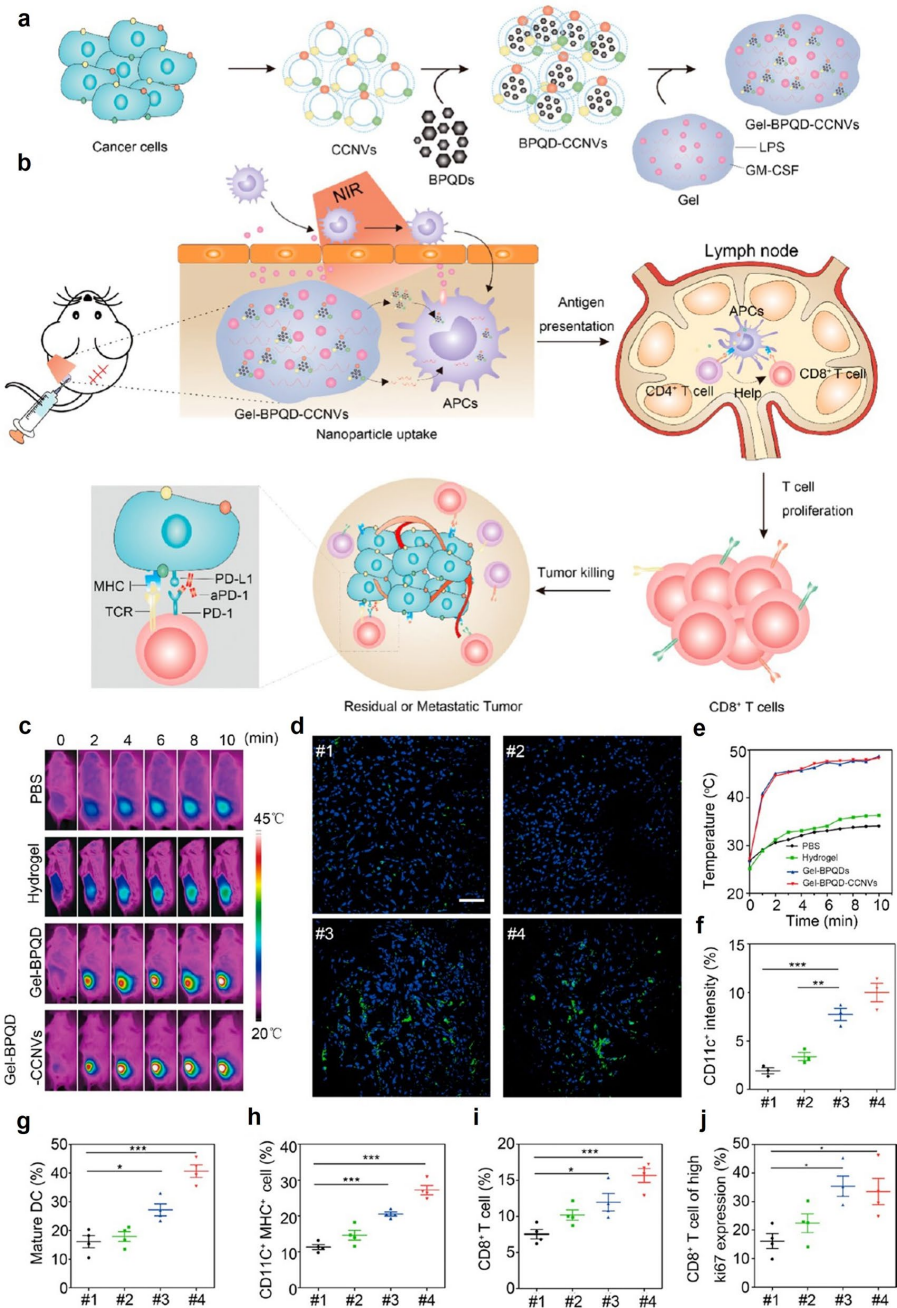
**a** Preparation of the BP-incorporated vesicles and their integration into hydrogel matrix. **b** Therapeutic synergism of the photothermal-capable hydrogel and PD-L1 antibody for the postoperative treatment of resected solid tumors. **c**/**e** IR thermal imaging and quantitative results showing the hydrogel-mediated local heating effects under NIT illumination. **d**/**f** Immunofluorescence and quantitative results of showing CD11 + DC infiltration in skin after hydrogel treatment. **g**-**j** Stimulatory effect of the hydrogel on various immune cell populations in tumor-draining lymph nodes. Reproduced with permission from Ref [89]. Copyright © 2019, American Chemical Society

The photothermal effect of BP-based nanomaterials is not only a direct tumor damaging modality but could also be exploited to regulate and amplify the therapeutic potency of other antitumor treatments. For instance, Xue et al. reported that doxorubicin (DOX), a first-line antitumor antibiotics, could be installed onto the surface of folic acid-modified BP nanosheets via physical absorption and released by NIR-mediated photothermal heating [88]. The DOX-loaded BP nanosheets (BP@DOX/PEG-FA) were further incorporated into gelatin/polycaprolactone (GP) hydrogel scaffold via electrospinning. The GP hydrogel is a bioresorbable material that may temporarily protect the BP nanosheets from various degradative factors after implantation and eventually be decomposed to eliminate long term safety concerns. Under NIR illumination at 808 nm, the BP-mediated photothermal effect would trigger the gel-sol transition of the BP substrate and release the BP@DOX/PEG-FA nanosheets into the ambient environment, which would be further taken in by residual tumor cells with upregulated folate receptors for combinational photothermal/chemotherapy. In addition to the tumoricidal effects, the degradation of the BP substrates would also release abundant phosphate/phosphonate ions into the tumor margin and activate the ERK1/2 and PI3k/Akt signaling pathways in skin cells at the wound region, which could promote their differentiation and proliferation to accelerate wound healing. Alternative to the introduction of chemotherapeutic components, Ye et al. reported a BP-based nanovaccine for combinational photothermal/immunotherapy of tumors. To prepare the dual-functional nanovaccine, BP quantum dots were first coated with the cytoplasmic membrane of surgically extracted tumors to afford nanovesicles, which were further loaded into thermosensitive hydrogel constituted by hyaluronic acid and pluronic F-127 (Fig. 3) [89] .During the preparation process, immune stimulative GM-CSF and lipopolysaccharide (LPS) were also loaded into the hydrogel by simple mixing. The composite hydrogel could be easily implanted into the resected tumor site via subcutaneous injection and gradually release the BP-incorporated nanovesicles, GM-CSF and LPS into the wound bed. The tumor cell membrane-coated nanovesicles were then captured by antigen-presenting cells (APCs) such as dendritic cells and macrophages for recognition of the tumor-derived neoantigens on the nanovesicle surface. The BP-mediated photothermal heating also enhanced the expression of MHC-I, MHC-II, CD80 and CD86 on APC surface, which may cooperate with the immunostimulatory functions of GM-CSF and LPS to promote the maturation of the APCs. The mature APCs would then migrate to the tumor-draining lymph nodes to activate tumor-specific effector T cells via cross-presentation. Interestingly, the BP-mediated photothermal effect could also breakdown the physical barriers in the residual tumor tissues by increasing blood flow, elevating vascular permeability and alleviating interstitial fluid pressure to facilitate the homing of activated T cells for efficient tumor elimination. The photothermal effect of BP quantum dots has also been used by Shou et al. to regulate the release of zoledronate from porous thermoresponsive poly(N-isopropylacrylamide) hydrogel to elicit γδ T cell-mediated antitumor responses. [90] Overall, these studies collectively demonstrate that the photothermal capability of BP-based materials potentiates many novel therapeutic opportunities for postoperative care of solid tumors via direct thermal ablation, drug release control and tissue environment remodeling.

**Fig. 4:**
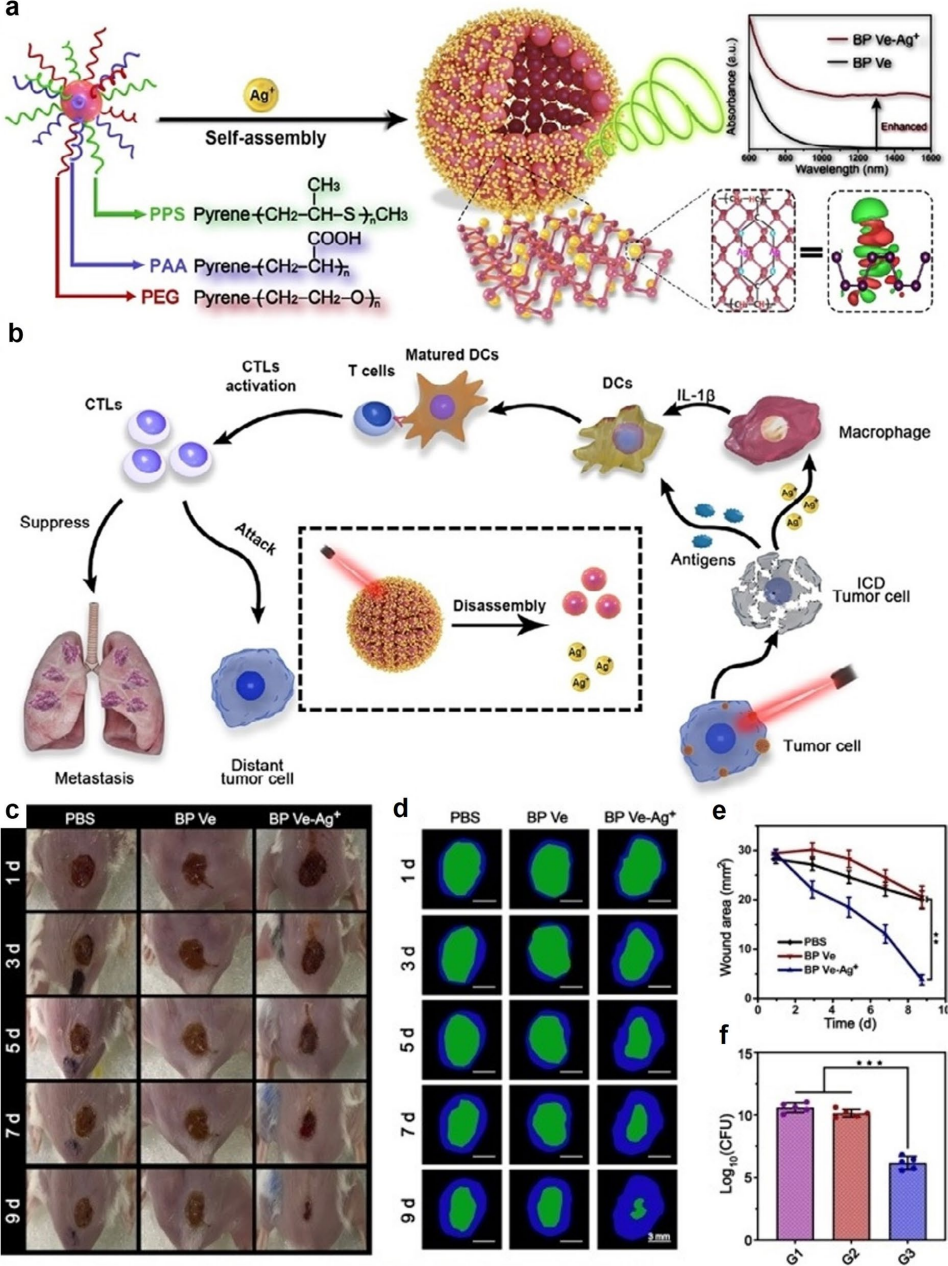
**a** Synthesis scheme of the Ag^+^ doped polymer-functionalized BP nanosheets and the self-assembly process into aqueous stable vesicles. **b** Therapeutic mechanism of the composite vesicle for combinational photodynamic/immunotherapy of resected solid tumors as well as the prevention of local bacterial infection. **c** Photographs of the *E. coli*-infected wounds after different treatment. **d** Changes in wound boundaries after different treatment. Blue area indicates original wounds, while green area indicates wounds after specific treatment for different days. **e** Changes in wound areas after different treatment. **f** Changes in the number of bacteria in the wound area after different treatment with PBS, BP Ve AND BP Ve-Ag^+^. Reproduced with permission for Ref [96]. © 2020 Wiley-VCH GmbH

In addition to their photothermal conversion capability, BP nanosheets could also absorb the photons in the wavelength range of around 660 nm and transfer the energy to ambient oxygen molecules to generate singlet oxygen [91–94]. Quantitative analysis further demonstrates that BP nanosheets have a quantum yield of around 0.91 in aqueous environment, substantiating its potential application as an efficient photosensitizer for photodynamic therapy [95]. For instance, Li et al. grafted three types of functional polymers onto BP quantum dots including polyethylene glycol (PEG), polyacrylic acid (PAA) and polypropylene sulfide (PPS), which could self-assemble into nanovesicles for combinational photodynamic/immunotherapy and enhance wound healing in resected solid tumors (Fig. 4) [96]. The PAA segments could be used for the anchoring of Ag^+^ ions that could reduce the band gap of BP quantum dots via charge coupling and thus enhance the photoacoustic signal for deep tissue imaging, while the ROS-responsive PPS segments could stabilize the composite BP-based nanosystem to reduce BP oxidation and Ag^+^ leakage during blood transportation. The BP-mediated PDT effect under NIR light would trigger the immunogenic death of tumor cells as well as removing the PPS segment to disassociate the nanovesicle, leading to the release of singular BP quantum dots and Ag^+^ ions. The Ag^+^ ions could stimulate the immune functions of macrophages and cooperate with the tumor-derived antigens released after PDT to elicit robust antitumor immunity. Moreover, the release Ag^+^ ions have high binding affinity with the sulfur groups on bacterial membrane to prevent wound infection. Notably, hypoxia is a hallmark of the tumor microenvironment that serious impedes the BP-mediated generation of singlet oxygen under NIR irritation [97–99]. To address this issue, Liu et al. conjugated manganese dioxide nanoparticles on the surface of BP nanosheets via electrostatic absorption, which could be decomposed in the acidic tumor lysosomes to release oxygen and support the PDT activity of BP nanosheets [100]. Yang et al. immobilized catalase-like platinum nanoparticles onto BP nanosheets to decompose the endogenous H_2_O_2_ for oxygen replenishment [101]. These emerging nanotechnologies offer new opportunities to amplify the antitumor potency of BP-mediated PDT in a clinical context.

As demonstrated by the discussions above, BP-based nanomaterials could simultaneously inhibit the growth of tumor cells while promoting the regeneration of damaged soft tissues under NIR illumination, and these properties are highly desirable for the postoperative care of resected tumors located in soft organs and tissues. Moreover, the intrinsic degradability of BP nanomaterials and the non-toxicity of the degradation products ensure that the BP-containing biomaterials could be gradually eliminated from the body, enhancing their short-term and long-term safety on real-life patients.

### Applications on bone-residing tumors

Bones are a group of highly specialized connective tissues in human body with a series of distinct anatomical and physiological features [102, 103]. From a general perspective, the bone tissue could be described as a rigid matrix of collagen and calcium phosphate, which is embedded with various types of cells such as osteoblasts, osteoclasts, osteocytes and bone lining cells [104–106]. Due to their superior mechanical strength and unique chemical composition compared to soft tissues such as fat, muscle and internal organs, bones perform multiple critical activities in human body including realization of body movement, protection of soft tissues and organs, and maintenance of calcium/phosphate homeostasis [107, 108]. Importantly, malignant tumors may also occur in bone tissues either through bone tumorigenesis or bone metastasis [109]. Surgical resection is the primary approach for treating bone tumors, during which the abnormal bones and affected soft tissues in the peripheral region are extensively excised. However, considering the important roles of bones in maintaining various physical and physiological functions of the human body, surgical removal of bone tumors would cause permanent disabling injuries as well as severe postoperative complications. Consequently, inhibiting the residual bone tumor cells while restoring the damaged bones is highly desirable for patients after bone tumor resection. However, the unique bone metabolism patterns create additional challenges for the repairing of the surgery-induced bone defects compared to damaged soft tissues. On one hand, the bone mass is maintained by the balance between the bone-forming functions of osteoblasts and bone-resorbing functions of osteoclasts, and it is thus often necessary to stimulate the activity of osteoblasts to rebuild the resected bone tissues [110]. On the other hand, to sustain the effective osteoblast-mediated bone healing would require abundant and continuous supply of bone-related nutrients, especially phosphate ions and calcium (Table 4).

**Table 4:** Summary on the preparation and therapeutic activity of various BP-based preparations for restoration of damaged bone tissues

Carrier substrate	Therapeutic additives	Bone healing mechanisms	Refs.
No	No	Enhancing ALP activity in human mesenchymal stem cells to promote their osteogenic differentiation	[115]
PLGA shell	Sr^2+^ ions	PTT-triggered Sr^2+^ release, phosphonate/phosphate supplementation	[118]
Hydroxyapatite scaffolds	Zn^2+^ ions	PTT-triggered Zn^2+^ release, phosphonate/phosphate supplementation, Zn-sensitized antibacterial effect	[119]
PLLA electrospun fibrous scaffold	BMP2	BP-enhanced biomineralization, BMP2-enhanced recruitment of osteoblast precursor cells	[120]
Mg implants with BP and PLGA double coating	dexamethasone	Dexamethasone-enhanced osteogenic effects	[121]
Aptamer-functionalized matrix vesicles	No	Aptamer-mediated osteoblast targeting effects, BP-enhanced biomineralization	[126]
PLLA nanofibrous scaffold	HA-SiO_2_ nanoparticles	Porous structures enable migration and infiltration of bone marrow-derived mesenchymal stem cells, PTT-induced release of bone nutrients	[127]
ECM-mimetic hydrogel	Calcium phosphate nanoparticles	PTT-induced release of bone nutrients	[128]
Gelatin-arginine hydrogel	No	Sustained P supplementation	[129]

Interestingly, photothermal-capable BP-based nanomaterials present multiple features that are highly relevant for guiding and enhancing bone regeneration to repair the postoperative bone defects. Typically, it is well-established that heating could enhance the alkaline phosphatase (ALP) activity and upregulate heat shock proteins (HSPs), which may facilitate the generation of seed crystals at the defect site and promote new bone formation [111–114]. Consequently, it is anticipated that the BP-mediated photothermal effect could fulfill the dual role of simultaneous tumor inhibition and bone healing. For instance, Raucci et al. reported that two-dimensional black phosphorus nanosheets obtained via liquid exfoliation method are well-tolerated by human mesenchymal stem cells (hMSCs) and also capable of substantially enhancing the ALP activity therein under NIR illumination, indicating that BP could promote the osteogenic differentiation of hMSCs to enhance new bone formation [115]. Meanwhile, the authors discovered that the BP nanosheets inhibited the growth of SAOS-2 osteosarcoma cells even without NIR irritation by downregulating the ALP expression in SAOS-2 cells to block their metabolic activities. Moreover, the BP nanosheets significantly downregulated the secretion of pro-inflammatory IL-6 cytokine while upregulating anti-inflammatory IL-10 secretion in the co-culture system of SAOS-2 and healthy osteoblast cells under NIR illumination, thus alleviating the osteosarcoma-induced inflammatory responses. The data collectively supported the utility of BP nanomaterials for the postoperative care of resected osteosarcoma by inhibiting residual osteosarcoma cells while activating the ALP-mediated bone regeneration pathways.

**Fig. 5:**
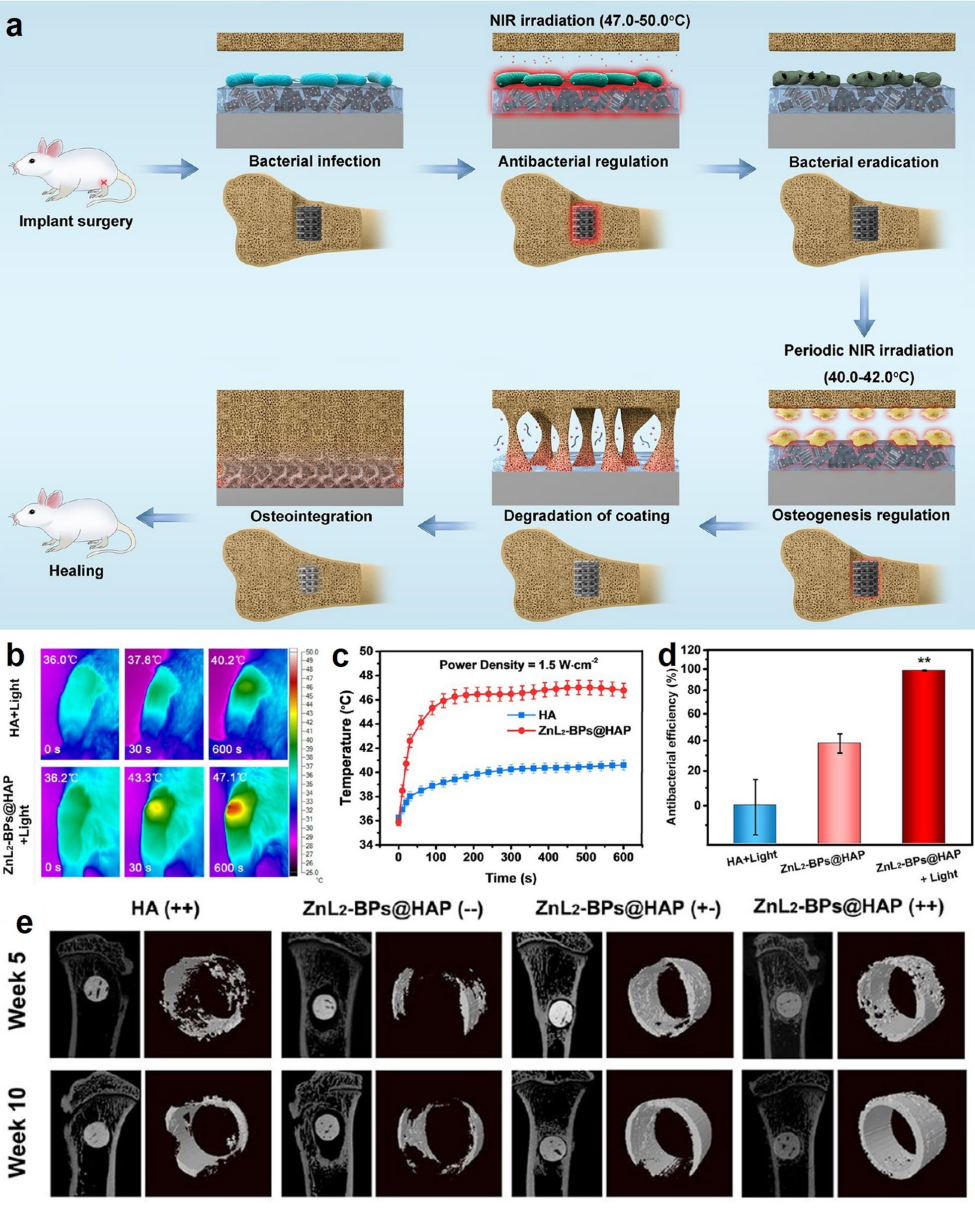
**a** Schematic illustration on the enhanced antibacterial and pro-osteogenic capability of hydroxyapatite scaffold modified with Zn^2+^-doped BP nanosheets. **b**/**c** Thermal imaging and temperature changes of the hydrogel implantation site in mice under NIR treatment. **d** Antibacterial performance of the BP-containing hydrogel with or without NIR treatment. **e** Micro-CT images of new bone formation around the implanted BP-containing hydrogel in vivo. Reproduced with permission from Ref [119]. Copyright © 2021, American Chemical Society

Instead of exploiting the BP-mediated photothermal effect as a remotely controllable trigger to regulate the bone regeneration-related signaling pathways, the versatile drug loading capability of BP-based nanomaterials has also attracted significant interest for both antitumor and bone-healing applications, which allows the efficient delivery of various bioactive substances to the surgical site and release them in a controlled manner. For instance, it has been reported that the long pair of electrons of the phosphorus atoms in the BP nanosheets could coordinate with divalent metal ions such as Mg^2+^, Zn^2+^, Sr^2+^ or Cu^2+^, which could activate the calcium-sensing receptors in bone-related cells to promote new bone formation [116, 117]. Typically, Wang et al. coated Sr^2+^-doped BP nanosheets with PLGA shell and used the resultant microparticles to enhance bone regeneration under osteoporotic conditions [118]. Under NIR irritation, the BP-mediated photothermal effect could trigger the release of Sr^2+^ ions to stimulate osteoblast differentiation while inhibiting bone resorption, which substantially augmented the bone regeneration at defect site in vivo. Based on similar metal ion-loading and releasing mechanisms, Wu et al. coordinated BP nanosheets with zinc sulfonate ligands and accommodated them on the surface of hydroxyapatite scaffolds, which could release Zn^2+^ ions in a controlled manner under NIR illumination (Fig. 5) [119]. The released Zn^2+^ ions could not only promote local osteogenesis but also enhance the photothermal susceptibility of peri-implant bacteria through inducing envelope stress, thus minimizing the risk of post-implantation infection. In addition to metal ions, BP nanosheets could also be used as the carrier substrate for small-molecule bone regeneration stimulants such as bone morphogenetic protein 2 (BMP-2), therapeutic peptides and dexamethasone [120–122]. Nevertheless, it is worth noting that bone tumor is a highly complex disease and the potential impact of these bone regeneration stimulants on tumor cells is still poorly understood. Consequently, more rigorous investigations regarding the safety and efficacy of BP-based photothermal responsive drug delivery systems are warranted under clinically relevant conditions, which may benefit the postoperative treatment of resected bone tumors.

**Fig. 6:**
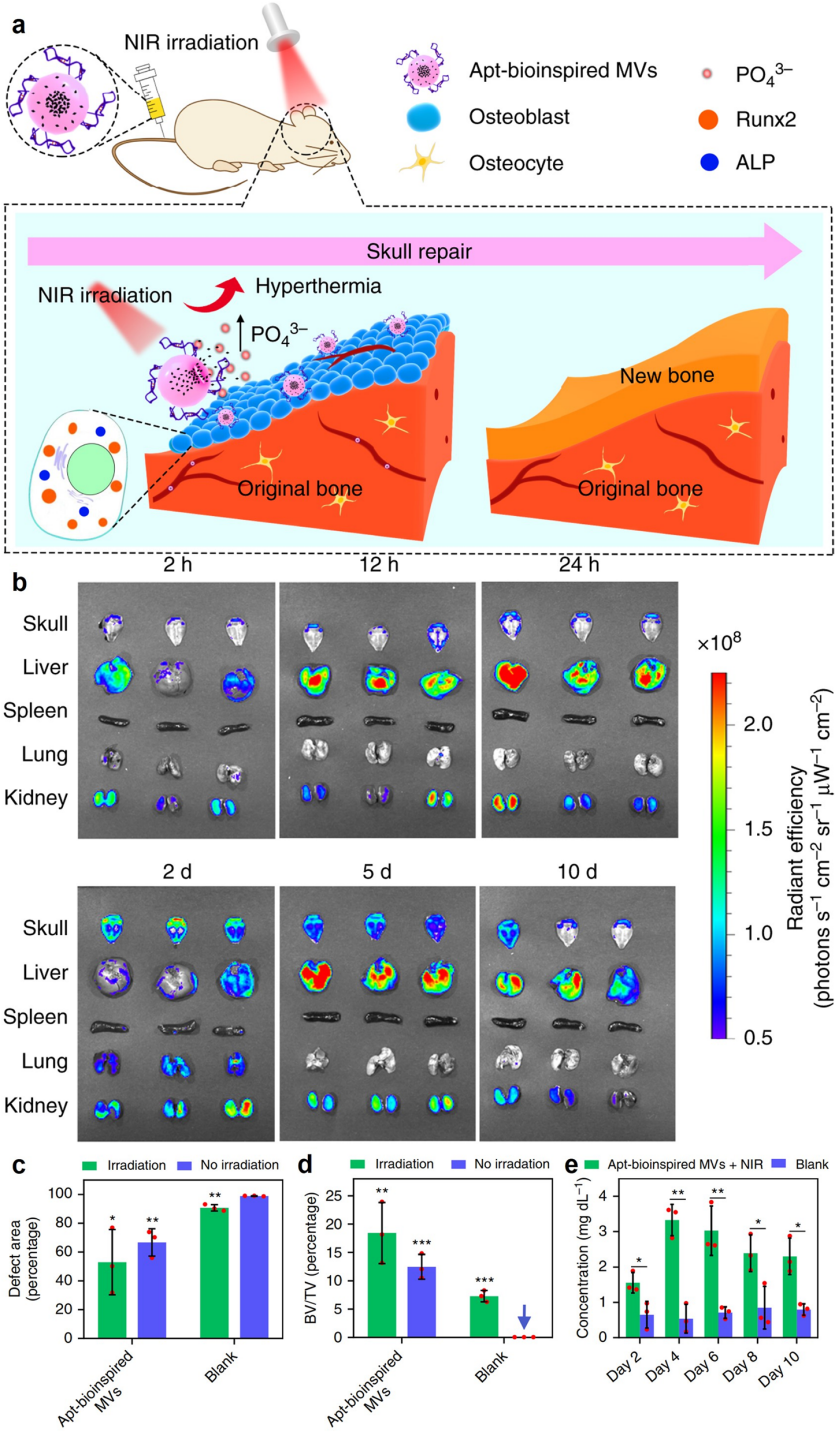
**a** Schematic illustration of the osteoblast-targeted BP-incorporated matrix vesicles to promote bone regeneration at defect site through NIR triggered release of PO_4_^3−^ ions and stimulation of biomineralization-related signaling pathways. **b** In vivo bone targeting effect of the BP-incorporated matrix vesicles. **c**/**d** Variations in the bone defect area and bone volume fraction after different treatment. **e** Time dependent changes of phosphate ion concentration in the bone defect area after different treatment. Reproduced with permission from Ref [126]. Copyright © 2019, The Author(s)

Hydroxyapatite (HA), the major mineral in mature bones, is a highly crystalline form of calcium phosphate [123, 124]. Consequently, bones act as the major reservoir site for phosphate in human body and play important roles in maintaining the homeostasis of phosphorus metabolism, while the regeneration of defective bones would require large amount of phosphate to support the biosynthesis of hydroxyapatite [125]. Interestingly, BP nanosheets could be easily oxidized into phosphate ions in physiological environment under NIR irritation, which not only enables controllable degradation of the implanted BP-based biomaterials but also reinforces the phosphate pool, potentially accelerating the bone regeneration process. Wang et al. loaded BP nanosheets into matrix vesicles (MVs) and further modified the MV surface with osteoblast-targeting aptamers (Fig. 6) [126]. After the intravenous injection into rabbits bearing artificially created skull defects, the BP-loaded MVs predominantly accumulated into the defect site due to the targeting effect of the aptamers and released abundant phosphate into the ambient environment under NIR irritation through the decomposition of the BP substrates, thus enhancing the formation of calcium phosphate minerals and promoting the biomineralization of osteoblasts in the skull defects. Li et al. developed a microfluidic strategy to synthesize a BP-based 3D nanocomposite fibrous scaffold, which is composed of HA-coated porous silica nanoparticles, BP nanosheets and 3D poly (l-lactic acid) (PLLA) nanoscaffold. The BP-mediated photothermal effect under NIR illumination could trigger the release of phosphate, calcium and silicon ions into the bone defect area and boost bone regeneration [127]. Similar effects have also been reported by Wang et al. and Huang et al., substantiating the potential application of BP-based nanomaterials as a phosphate supplier to accelerate new bone formation [128, 129].

## Perspectives and conclusion

BP nanosheet is an emerging 2D inorganic nanomaterial with many unique and exciting capabilities, which shows promise to revolutionize various fields in biomedical technology. Specifically, BP nanosheets possess a direct and thickness-dependent band-gap rarely found in other typical 2D materials, which is correlated to a wide light absorption range from visible light to infrared range. The unique optoelectrical properties of BP nanosheets make them an exceptional candidate for many biomedical applications such as biosensing, in vivo imaging and light-dependent therapies. Meanwhile, BP nanosheets also presents large surface-to-volume ratio, high anisotropic structural and functional properties and inherent bioactivity, which prompts their application not only as an efficient drug carrier, but also a versatile platform for the realization of novel therapeutic concepts including chemotherapy, photothermal/photodynamic therapy, immune therapy, radiotherapy, bioactive therapy and combinational therapies. Furthermore, BP nanosheets are completely degradable in biological milieu by reacting with oxygen and water molecules, of which the degradation products are non-toxic and can readily join the endogenous phosphorus metabolism to meet the increasing demand for nutrients during the regeneration of both solid and hard tissues. These inherent and functional merits have tremendously spurred research and application of BP-based biomaterials in almost all fields of biomedical technology and substantiated their extraordinary potential for postoperative tumor treatment.

Nevertheless, it is worth noting that the research and development of BP-based biomaterials are still in an early stage compared to other well-studied 2D inorganic nanomaterials such as graphene and MXenes, as there are many issues and challenges regarding their synthesis, modification and implementation that remain to be solved. Currently, few-layer BP nanosheets are predominantly synthesized by time-consuming exfoliation method with limited yield, and the product quality is largely determined by the bulk BP crystal seeds [130]. As the photoelectrical and biological properties of BP nanosheets are profoundly affected by their morphological characteristics including layer numbers, size, shapes and structural defects, it is important to develop new BP synthesis technologies with enhanced topological control and higher production yield. On the other hand, BP nanosheets are prone to oxidation-dependent degradation in biological milieu. Although the spontaneous degradation behavior of BP may help to reduce the long-term health risks of the treatment while generating phosphate ions necessary for tissue regeneration, it may also potentially impair the therapeutic persistence for prolonged treatment of postoperative wounds as well as causing acute phosphate toxicity. It is therefore important to balance the therapeutic efficacy and degradation rate of BP nanosheets via proper surface modification and stabilization. Furthermore, despite the relatively low cellular and systemic toxicity of BP-based biomaterials demonstrated in various pre-clinical studies, their toxicity profiles in human body still haven’t been comprehensively studied, which severely restrict the clinical translation of BP-based biomaterials. With the continuous progress in both the fundamental and applied aspects of BP biomaterials, we are confident that this burgeoning technology could be exploited as an efficacious and safe adjuvant therapy for tumor surgery with clinical promise.

## Data Availability

Not applicable.
